# Emerging role of HDAC11 in skeletal muscle biology

**DOI:** 10.3389/fcell.2024.1368171

**Published:** 2024-05-27

**Authors:** Jihong Chen, Qiao Li

**Affiliations:** ^1^ Department of Pathology and Laboratory Medicine, Faculty of Medicine, University of Ottawa, Ottawa, ON, Canada; ^2^ Department of Cellular and Molecular Medicine, Faculty of Medicine, University of Ottawa, Ottawa, ON, Canada

**Keywords:** histone acetylation, histone deacetylase, gene regulation, chromatin modification, myogenic differentiation

## Abstract

HDAC11 is an epigenetic repressor of gene transcription, acting through its deacetylase activity to remove functional acetyl groups from the lysine residues of histones at genomic loci. It has been implicated in the regulation of different immune responses, metabolic activities, as well as cell cycle progression. Recent studies have also shed lights on the impact of HDAC11 on myogenic differentiation and muscle development, indicating that HDAC11 is important for histone deacetylation at the promoters to inhibit transcription of cell cycle related genes, thereby permitting myogenic activation at the onset of myoblast differentiation. Interestingly, the upstream networks of HDAC11 target genes are mainly associated with cell cycle regulators and the acetylation of histones at the HDAC11 target promoters appears to be residue specific. As such, selective inhibition, or activation of HDAC11 presents a potential therapeutic approach for targeting distinct epigenetic pathways in clinical applications.

## Introduction

Histone deacetylases (HDACs) are evolutionarily conserved and often found at transcriptionally inactive loci and heterochromatins (Vaquero et al., 2004; Wang et al., 2009). They act through their deacetylase activity to remove functional acetyl groups from the lysine residues of histones as well as non-histone proteins. A total of 18 HDACs have been found in mammals, classified as the Zn^2+^-dependent class I, II, IV, and the NAD^+^-dependent class III HDACs (Park and Kim, 2020). HDAC11 is the sole member of the class IV HDACs (Gao et al., 2002). Besides the deacetylase property, it also contains potent fatty deacylation and lysine demyristoylation activities (Kutil et al., 2018; Moreno-Yruela et al., 2018; Cao et al., 2019; Bagchi et al., 2022). While HDAC inhibition has been employed as a therapeutic approach for cancer treatments (Bondarev et al., 2021), increasing evidence suggests that HDACs can also be targeted selectively for the treatment of other diseases, including muscle related diseases.

### HDAC11 in immune response

HDAC11 has been implicated in the regulation of different immune cells and cellular responses. It represses IL-10 gene expression through a direct control of promoter accessibility in antigen-presenting cells (Villagra et al., 2009; Cheng et al., 2014). Upregulation of HDAC11 expression through the inhibition of microRNA-145 by type I interferon signaling decreases innate IL-10 production in macrophages (Lin et al., 2013). HDAC11 is also a negative regulator of the expansion and function of myeloid derived suppressor cells (Sahakian et al., 2015) and affects myeloid differentiation such as chemokine and cytokine expression during neutrophil maturation, migration, and phagocytic function (Sahakian et al., 2017). It plays a role in T cell development and tumor biology (Buglio et al., 2011; Huang et al., 2017; Woods et al., 2017; Bora-Singhal et al., 2020; Wang et al., 2020). Interestingly, the deacylation activity of HDAC11 has also been implicated in the regulation of type I interferon signaling (Cao et al., 2019).

### HDAC11 in metabolic pathway

HDAC11 has been shown to play a regulatory role in metabolic homeostasis. For example, *Hdac11* knockout mice exhibit better metabolic health and less susceptible to high-fat diet induced weight gain (Sun et al., 2018). Ablation of *Hdac11* improves insulin sensitivity and glucose tolerance, in addition to boosts energy expenditure though promoting thermogenic capacity (Sun et al., 2018). The benefit of Hdac11 deficiency is associated with an increased uncoupling protein one expression and an elevation of brown adipose tissue abundance and activity, but beiging of white adipose tissue (Bagchi et al., 2018; Sun et al., 2018). Mechanistically, Knockdown of *Hdac11* promotes brown adipocyte differentiation and attenuates the suppressive role of Hdac11 on thermogenic program of adipose tissue that is dependent on its physical association with BRD2, a bromodomain and extraterminal acetyl-histone-binding protein (Bagchi et al., 2018). In addition, Hdac11 has been implicated in adrenergic signaling pathway (Bagchi et al., 2022). In skeletal muscle, Hdac11 depletion increases muscle strength and fatigue resistance through AMP-activated protein kinase-acetyl-CoA carboxylase signaling pathway to enhance mitochondrial fatty acid β-oxidation (Hurtado et al., 2021).

### HDAC11 in cell cycle regulation

Chromatin reorganization is important for the processes of DNA replication and cell cycle progression. HDAC11 physically interacts with and deacetylates replication licensing factor Cdt1 (Glozak and Seto, 2009). It is upregulated in several models of renal fibrosis, promoting pro-fibrogenic response likely through the repression of Kruppel-like factor 15 gene expression (Mao et al., 2020). HDAC11 is also overexpressed in several carcinomas, and depletion of HDAC11 decreases cancer cell metabolic activity and viability through apoptosis (Deubzer et al., 2013). A group of cell cycle promoting genes regulated by HDAC11, essential for tumor cell viability, has been identified in neuroblastoma related models, suggesting a regulatory role for HDAC11 in mitotic cell cycle progression and cell division (Thole et al., 2017). In fibroblasts, the level of HDAC11 is low in cycling cells but high in quiescence cell, and overexpression of HDAC11 inhibits cell cycle progression of both transformed and nontransformed cells (Bagui et al., 2013). Likewise, depletion of Hdac11 upregulates cell cycle related genes in skeletal myoblasts (Byun et al., 2017; Núñez-Álvarez et al., 2021). Nevertheless, the molecular pathways by which HDAC11 affects cell cycle progression in myogenic differentiation remains unclear.

### HDACs in skeletal muscle development

Adult muscle regeneration is an important physiological process to maintain muscle homeostasis and repair the muscle following injury. The regenerative responses are mediated by resident muscle stem cells (MuSCs) or the satellite cells (Cornelison et al., 2001; Seale et al., 2004; Yin et al., 2013), a population of stem cells found between the myofiber sarcolemma and the basal lamina. Characterized by the expression of the Paired-box protein 7 (PAX7) (Mauro, 1961; Dumont et al., 2015), MuSCs are quiescent in healthy muscle, become activated to proliferate and differentiate into new myofibers upon muscle injury or exercise, but can also revert to the quiescent state to maintain the MuSC pool (self-renewal) for future regeneration (Giordani et al., 2018). Mechanistically, muscle regeneration is a multistage event, consisting of myoblast proliferation, differentiation, and myocyte fusion, which is tightly controlled by different myogenic regulatory factors (MRFs), including MyoD and myogenin (Tapscott, 2005). On a molecular level. myogenic differentiation begins with the downregulation of PAX7 and upregulation of MRFs which coordinate the commitment, terminal differentiation, and fusion into myofibers (Berkes and Tapscott, 2005; Blais et al., 2005; le Grand and Rudnicki, 2007; Chang and Rudnicki, 2014; Conerly et al., 2016). In early myoblast differentiation, residue-specific histone acetylation signifies the regulatory loci concerted by MRFs and histone acetyltransferase (HAT) p300 (Hamed et al., 2013; 2017; Khilji et al., 2018; 2020; 2021).

On the other hand, many studies have demonstrated the impact of HDACs on muscle development. Deacetylation of MyoD by HDAC1 silences MyoD-mediated gene expression (Mal, 2001; Mal and Harter, 2003). The Snai1-HDAC1/2 repressive complex excludes MyoD from differentiation-specific regulatory elements in proliferating myoblasts, preventing the entry into myogenic differentiation (Soleimani et al., 2012). HDAC4 interacts with the transcription factor MEF2 and deacetylates myosin heavy chain, exerting a regulatory role in both myogenic differentiation and muscle homeostasis (Lu et al., 2000; Marroncelli et al., 2018; Luo et al., 2019). In addition, HDACs are required for heterochromatin reorganization during terminal myoblast differentiation (Terranova et al., 2005). Interestingly, it has been shown that HDAC11 is expendable for muscle stem cell formation and adult muscle growth, while knockout of *Hdac11* in mice encourages muscle regeneration following muscle injury (Byun et al., 2017; Núñez-Álvarez et al., 2021).

### HDAC11 in early myoblast differentiation

HDAC function is required for histone deacetylation to suppress target gene expression which is essential for stem cell fate transition. In early myoblast differentiation, *Hdac11* is the most significantly upregulated HDACs and change in residue-specific histone acetylation occurs at the promoters of differentially expressed genes (Li et al., 2023). In addition, the Hdac11 gene locus is controlled by histone acetyltransferase (HAT) p300 and the muscle master regulator MyoD (Li et al., 2023), and ablation of *Hdac11* in mice results in persistent myoblast proliferation in culture, albeit the induction signal for differentiation (Núñez-Álvarez et al., 2021).

**FIGURE 1:**
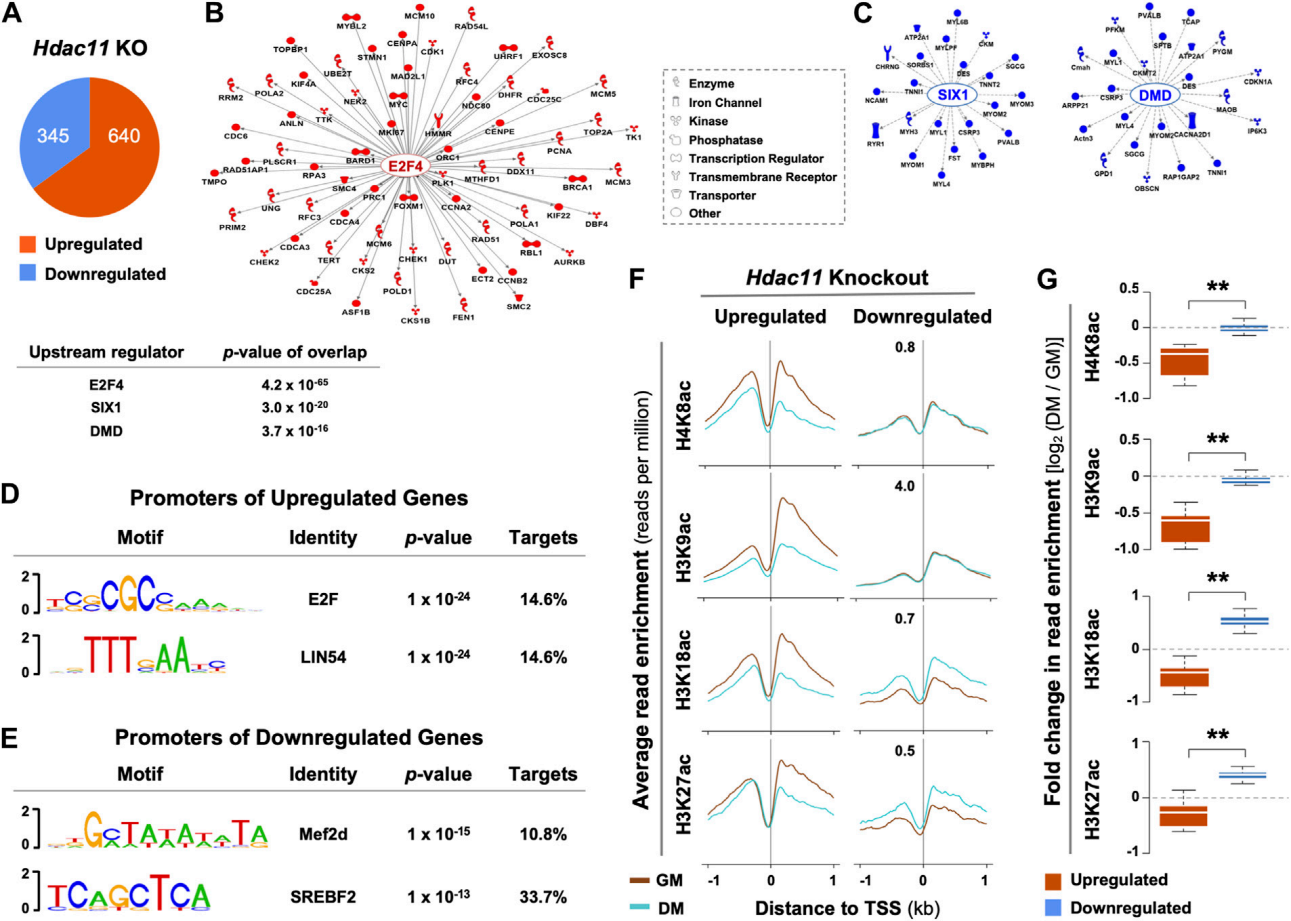
Upstream regulatory pathways and histone acetylation at the promoters of genes impacted by *Hdac11* ablation. **(A)** Hypergeometric Optimization of Motif EnRichment (HOMER) was used to annotate genes which were significantly upregulated or downregulated upon *Hdac11* knockout in mice (KO, ≥ ±1.5 absolute fold change, GSE147423). **(B)** QIAGEN Ingenuity Pathway Analysis was used for the upregulated genes as categorized in Panel **(A)**. The molecular network displays direct gene interactions, and the shapes of the nodes distinguish functional gene classes. **(C)** Network analysis for the downregulated genes. **(D)** HOMER was used for motif analysis of the promoters of genes upregulated following the *Hdac11* knockout. **(E)** Motif analysis of the promoters of genes downregulated. **(F)** The ngs.plot was used to visualize the average enrichment profiles of histone acetylation across the transcription start site (TSS, ±1 kb, GSE94558) in wild type differentiating (DM) or proliferating (GM) myoblasts, corresponding to the genes up- or downregulated upon the *Hdac11* knockout. **(G)** Quantification of log_2_-fold change in histone acetylation signals as in panel C (Wilcoxon rank sum test, **p < 2.2 × 10^−16^).

The effects of Hdac11 on myoblast proliferation appears to be mediated at the level of gene expression. Based on the public deposited RNA-seq data of the primary myoblasts isolated from the *Hdac11* knockout mice (Núñez-Álvarez et al., 2021), 640 genes were significantly upregulated by over 1.5-fold when compared to the wild-type controls, while 345 genes were downregulated (Figure 1A). Interestingly, Ingenuity Pathway Analysis (IPA) identified E2F4 as a prominent upstream regulator for 70 upregulated genes that are related to cell cycle and DNA replication processes (Figure 1B). On the other hand, SIX1 and DMD were the top two upstream regulators associated with downregulated genes that are mostly attributed to muscle system process in a smaller gene group (Figure 1C). Additionally, consensus binding sites for E2F and LIN54, known cell cycle regulators (Takahashi et al., 2000; Marceau et al., 2016), were found to be the top two binding motifs found in the promoters of genes upregulated by *Hdac11* ablation. (Figure 1D). In contrast, the top two motifs at the promoters of downregulated genes were best matched for Mef2d and SREBF binding (Figure 1E).

Based on the deposited histone acetylation ChIP-seq data from the wild type myoblasts (GSE94558), H4K8, H3K9, H3K18 and H3K27 acetylation at the promoters of genes upregulated by Hdac11 inactivation, corresponded to a decreased profile in the wild type differentiating myoblasts compared to undifferentiated controls. Conversely, the promoters of downregulated genes correlated with an increased acetylation at H3K18 and H3K27 in the wild type differentiating myoblasts (Figures 1F, G). The fact that the promoters of genes upregulated by *Hdac11* knockout were associated with decreased histone acetylation in normal myoblast differentiation supports the notion that HDAC11 deacetylates histones at these promoters to repress gene transcription.

**FIGURE 2:**
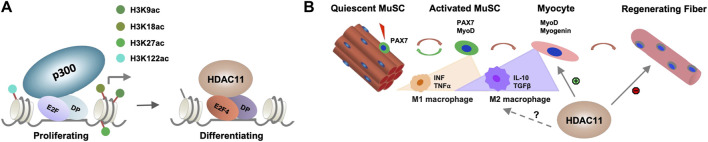
Molecular mechanisms of HDAC11 function. **(A)** Role of HDAC11 in cell cycle related genes. E2F related transcription factor and their dimerization partner (DP) recruit HDAC11 to the regulatory loci associated with p300 HAT in proliferating myoblasts for histone deacetylation to permit the onset of differentiation. **(B)** Potential impact of HDAC11 on immune response in muscle regeneration. During muscle regeneration, the progression of MuSC proliferation to differentiation is associated with a transition of M1 to M2 macrophages which secrete distinct cytokines within the muscle tissue. HDAC11 exerts positive or negative impact on muscle regeneration depending on distinct molecular pathway evoked.

While histone deacetylation is required for switching gene programs from proliferation to differentiation, HDACs do not directly bind to the DNA regulatory elements to repress gene transcription. The E2F family of transcription factors are key cell cycle regulators and can act as either transcriptional activators or repressor depending on cellular context (Takahashi et al., 2000). E2F promoted cell cycle progression critically depends on the function of p300 HAT which is antagonized by HDACs (Morris et al., 2000). Therefore, the interplay of HDAC11 and HAT may be essential for reversible histone acetylation at the target genes to permit stem cell fate transition, in that specific cell cycle regulators are responsible for recruiting HDAC11 to suppress cell cycle progression, which is a prerequisite for the initiation of myogenic differentiation (Figure 2A). Consequently, the downregulation of myogenic expression following Hdac11 ablation, may reflect the paucity of myogenic differentiation because of persistent cell cycle activity.

## Discussion

To permit myogenic differentiation, cell cycle arrests via histone deacetylation mediated gene repression. Like many other HDACs, HDAC11 is an epigenetic repressor of gene transcription attributed to its deacetylate activity. Although the upstream regulatory networks of Hdac11 target genes are associated with cell cycle regulators in differentiating myoblasts (Figure 1), transcription factors that recruit HDAC11 to the regulatory loci and Hdac11 associated histone deacetylation in myogenic differentiation remain to be determined. Future research with integrated approach of gene specific targeting coupled with omics analyses of transcriptome and genome wide protein-DNA interaction will allow to identify novel regulatory mechanisms associated with HDAC11 function and delineate the molecular basis of HDAC and HAT interplay in reversible histone acetylation during stem cell fate transition.

In addition, MuSC fate and homeostasis are also regulated by non-muscle cells in the muscle microenvironment and different immune cells form essential components of the MuSC niche upon muscle injury (Deng et al., 2012; Dinulovic et al., 2017). The transition of M1 to M2 macrophage is particularly critical to MuSC fate transition (Forcina et al., 2020). Given the described roles for HDAC11 in the regulation of different immune responses, the impact of HDAC11 on skeletal muscle development is no doubt multifaceted, muscle metabolism and growth, MuSC fate transition, and beyond (Figure 2B). As such, delineating the positive or negative role of HDAC11 involved in distinct steps of myogenic differentiation will help develop the best strategy to selectively inhibit or activate HDAC11 for muscle regeneration and repair in muscle therapeutics.

## Data Availability

The original contributions presented in the study are included in the article/Supplementary Material, further inquiries can be directed to the corresponding author.
